# Do children with neurological disabilities use more inpatient resources: an observational study

**DOI:** 10.1186/s12982-017-0059-1

**Published:** 2017-04-27

**Authors:** Jin-Xi Yuan, Marian McGowan, Irene Hadjikoumi, Buddhi Pant

**Affiliations:** 1 St George’s University of London, Cranmer Terrace, London, SW17 0RE UK; 2 910 Garratt Lane, London, SW17 0ND UK; 3 Child Development Centre, St George’s Hospital, Blackshaw Road, Tooting, London, SW17 0QT UK

**Keywords:** Neurodisability, Epidemiology, Paediatrics

## Abstract

**Background:**

Advances in healthcare have improved the survival of children with neurological disabilities (ND). Studies in the US have shown that children with ND use a substantial proportion of resources in children’s hospitals, however, little research has been conducted in the UK. We aimed to test the hypothesis that children with neurological disabilities use more inpatient resources than children without neurological disabilities, and to quantify any significant differences in resource use.

**Methods:**

A retrospective observational study was conducted, looking at the number of hospital admissions, total inpatient days and the reason for admissions for paediatric inpatients from January 1st to March 31st 2015. Inpatients were assigned into one of three groups: children without ND, children with one ND, and children with more than one ND.

**Results:**

The sample population included 942 inpatients (mean age 6y 6mo). Children with at least one ND accounted for 15.3% of the inpatients, 17.7% of total hospital inpatient admission episodes, and 27.8% of the total inpatients days. Neurological disability had a statistically significant effect on total hospital admissions (*p* < 0.001). Neurological disability also had a statistically significant effect on total inpatient days (*p* < 0.001). Neurological disability increased the length of inpatient stay across medicine, specialties, and surgery.

**Conclusions:**

Children with ND had more frequent hospital admission episode and longer inpatient stays. We identified a smaller group within this population, with arguably more complex neurological disabilities, children with more than one ND. This group had the highest number of admissions and longest inpatient stays. More frequent hospital admissions and longer inpatient stays may place children with ND at greater risk of the adverse effects of hospitalisations. We recommend further investigations looking at each the effects of the different categories of ND on inpatient resource use, and repeat of this study at a national level and over a longer period of time.

## Background

Advances in healthcare have improved the survival of children with neurological disabilities (ND) [1]. There has been a growing interest in the hospital resource use by children with ND. Studies in the US have shown that children with complex chronic conditions, of which includes children with ND, use a substantial proportion of resources in children’s hospitals [2], accounting for a growing proportional number of paediatric hospital days and hospital charges [3].

A recurring theme in the available literature is the presence of a subset within the disabled population that accounts for a large proportion of healthcare resources [3, 4]. Studies of cerebral palsy in Australia has linked higher degrees of severity and complexity to more frequent hospital admissions [5–7]. Some studies have equated a large amount of resource use by a small proportion of the hospital population, as being representative of a disproportional use of healthcare resources by children with ND [3, 8, 9].

In the UK, there is a general perception among healthcare professionals that children with ND use a disproportionate amount of inpatient resources. However, there is little research specifically looking at children with ND as a population group.

## Methods

This paper aims to test the hypothesis that children with ND use more inpatient resources, looking specifically at the number of hospital admissions and length of inpatient stay. Childhood disability is estimated to be around 7.3% (CI 6.9–7.7%), under the Disability Discrimination Act definition [10]. With this figure as an estimated baseline, we could compare inpatient resource use by children with and without ND in our sample hospital population.

We conducted a retrospective observational study for inpatients at three paediatric wards (one surgical and neuroscience ward, and two general medicine wards), at St George’s Hospital, London. The sample hospital population included all admitted paediatric inpatients who had an admission episode that included at least one day between January 1st and March 31st 2015.

Using this hospital population, for each patient we looked at:

- Number of admission episodes: where each episode included at least one day between January 1st and March 31st 2015.
- Length of each inpatient admission episode: where each episode included at least 1 day between January 1st and March 31st 2015.
- Total inpatient days: summation of inpatient admission episodes.
- Reason for admission: medicine, surgery or specialties.
- Neurological disability.

All inpatients were given a set of clinical codes based on current diagnosis, and any previous diagnoses including neurological disabilities. ND was split into 6 categories [11]: (1) intellectual and developmental disorders; (2) autism spectrum disorder; (3) cerebral palsy; (4) epilepsy; (5) genetic, chromosomal and syndromic conditions; and (6) more than one ND. Children with clinical codes that fell into only one of the first 5 categories were assigned “children with one ND”. For children with clinical codes in multiple categories, they were assigned “children with more than one ND”. Children without ND group were admitted mostly with acute illnesses such as a viral-induced wheeze, and chronic “non-ND conditions” such as sickle cell disease.

A one-way ANOVA, and Fisher’s LSD was used to compare the means of the three population groups for number of admission episodes and total inpatient days. A two-way ANOVA was performed comparing the effects of admission reason and neurological disability on the mean total inpatient days.

## Results

**Fig. 1:**
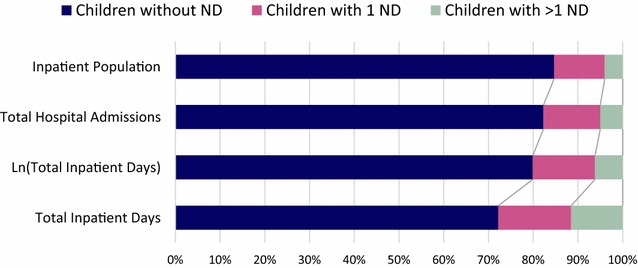
Proportion of inpatient resource use by children without ND, children with one ND and with more than one ND

The sample hospital population consisted of 942 inpatients with at least one admission episode that included one day between January 1st and March 31st 2015. The mean age 6 years 6 months (SD 5y 5mo). There were 798 children without ND (mean age 6y 0mo; SD 5y 4mo), 106 children with one identified ND (mean age 6y 6mo; SD 5y 1mo), and 38 children with more than one identified ND (mean age 7y 11mo; SD 5y 6mo). Figure 1 shows the proportion of inpatient resource use by population group.

### Total hospital admissions

A one-way ANOVA showed that the effect of ND on total hospital admission was significant, *F*(2,939) = 15.13, *p* < 0.001. Fisher’s LSD showed:

- The difference in admissions between children without ND and children with one ND was significant, *t*(939) = 4.02, *p* < 0.001.
- The difference in admissions between children without ND and children with more than one ND was significant, *t*(939) = 4.04, *p* < 0.001.
- The difference in admissions between children with one ND and children with more than one ND was not statistically significant, *t*(939) = 1.35, *p* > 0.05.

### Total inpatient days

A one-way ANOVA showed that the effect of ND on total inpatient days was significant, *F*(2,939) = 21.97, *p* < 0.001. Fisher’s LSD showed:

- The difference in total inpatient days between children without ND and children with one ND was significant, *t*(939) = 2.93, *p* < 0.003.
- The difference in total inpatient days between children without ND and children with more than one ND was significant, *t*(939) = 6.14, *p* < 0.001.
- The difference in total inpatient days between children with one ND and children with more than one ND was significant, *t*(939) = 3.79, *p* < 0.001.

The raw data was very positively skewed (Table 1), with many inpatients only admitted once during the study and recording very short admission episodes. To reduce the skewness, the natural logarithm of the *Total Inpatient Days* was taken, denoted by: *ln*(*Total Inpatient Days*). Using ln(Total Inpatient Days), a one-way ANOVA showed that the effect of ND on total inpatient days was significant, *F*(2,939) = 30.20, *p* < 0.001. Fisher’s LSD showed, using Ln(Total Inpatient Days)

**Table 1:** The mean, median, and skewness of *Total Hospital Admissions*, *Total Inpatient Days*, and *ln(Total Inpatient Days). The inverse values of ln (Total Inpatient Days) is shown*

	Mean (SE)	Median (IQR)	Skewness (SE)
Children without ND			
Total hospital admissions	1.10 (0.01)	1.00 (0.00)	5.00 (0.09)
Total inpatient days	5.51 days (0.40)	3 days (4 days)	10.38 (0.09)
ln(Total Inpatient Days)	3.67 days (0.03)	3 days (3 days)	1.18 (0.09)
Children with one ND			
Total hospital admissions	1.28 (0.06)	1.00 (0.00)	2.53 (0.24)
Total inpatient days	9.35 days (1.41)	5 days (7 days)	3.98 (0.24)
ln(Total Inpatient Days)	5.51 days (0.09)	5 days (3.33 days)	1.01 (0.24)
Children with more than one ND			
Total hospital admissions	1.39 (0.11)	1.00 (1.00)	1.50 (0.38)
Total inpatient days	18.47 days (4.62)	7 days (15 days)	2.52 (0.38)
ln(Total Inpatient Days)	8.31 days (0.20)	7 days (6 days)	0.59 (0.38)

- The difference in total inpatient days between children without ND and children with one ND was significant, *t*(939) = 5.01, *p* < 0.001.
- The difference in total inpatient days between children without ND and children with more than one ND was significant, *t*(939) = 6.29, *p* < 0.001.
- The difference in total inpatient days between children with one ND and children with more than one ND was significant, *t*(939) = 2.78, *p* < 0.006.

**Fig. 2:**
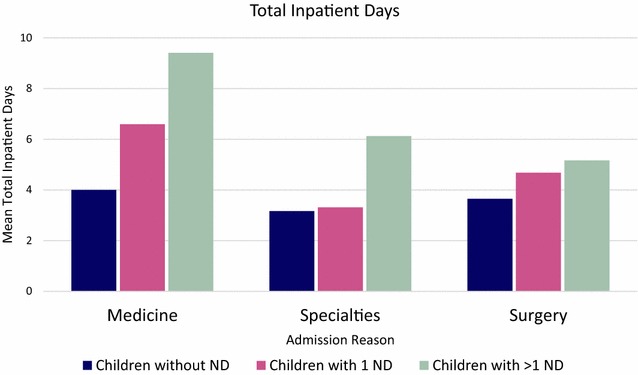
A comparison of the effect of *Neurological Disability* and *Admission Reason* on *ln(Total Inpatient Days)*. The inverse of the mean *ln(Total Inpatient Days)* is shown

A two-way ANOVA (Fig. 2) was performed looking at the effect of *Neurological Disability* and *Admission Reason* on the mean *ln*(*Total Inpatient Days*). Neurological disability had a significant main effect on inpatient days, *F*(2,933) = 9.31, *p* < 0.001. Admission reason also had a significant main effect on inpatient days, *F*(2,933) = 6.24, *p* < 0.002. There was no significant interaction between neurological disability and admission on inpatient days, *F*(4,933) = 1.65, *p* > 0.05.

### Neurological disability

A post hoc analysis was undertaken to test whether the category of ND had a significant effect on inpatient resource use (Table 2).

**Table 2:** Frequency of different ND categories

Neurological disability	Number of inpatients
Intellectual and development disorders	14
Autism spectrum disorder	7
Cerebral palsy	4
Epilepsy and seizures	13
Genetic, chromosomal, syndromes	68
More than one ND	38

**Fig. 3:**
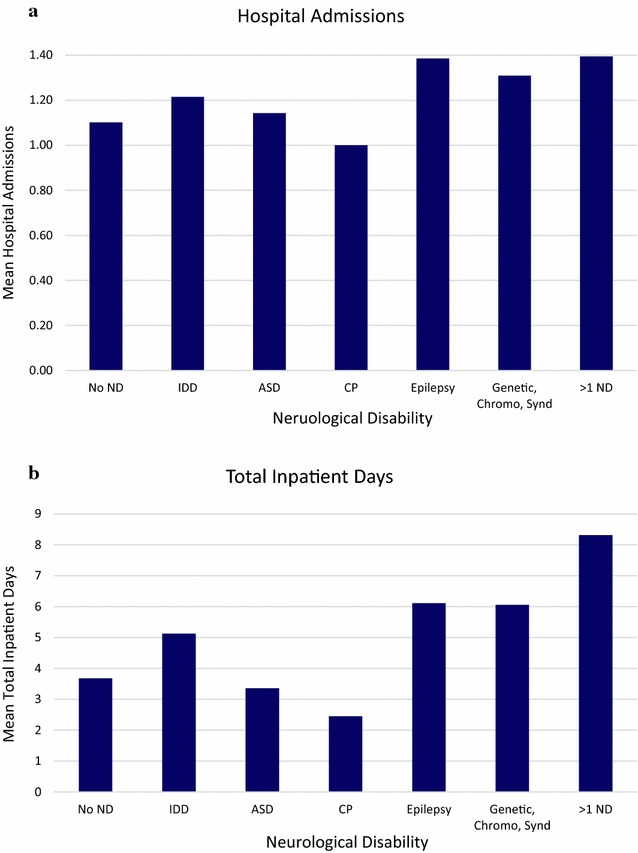
A comparison of the effect of different neurological disabilities on **a** mean hospital admissions, **b** total inpatient days

A one-way ANOVA showed that the effect of the category of ND on hospital admissions was significant (Fig. 3a), *F*(6,935) = 5.66, *p* < 0.001. A Tukey post hoc test revealed that those with more than one ND (+0.29 admissions, 95% CI 0.08–0.51, *p* < 0.001), and those with genetic, chromosomal and syndromic disorders (+0.21 admissions, 95% CI 0.04–0.37, *p* < 0.003), had statistically significantly more admissions than children without ND.

A one-way ANOVA showed that the effect of the category of ND on total inpatient days (using transformed data) was significant (Fig. 3b), *F*(6,935) = 11.54, *p* < 0.001. A Tukey post hoc test revealed that those with more than one ND (+2.26 days, 95% CI 1.54–3.31, *p* < 0.001) and those genetic, chromosomal and syndromic disorders (+1.65 days, 95% CI 1.23–2.20, *p* < 0.001), had statistically significantly more inpatient days than children without ND.

## Discussion

Our findings were consistent with the hypothesis that children with ND use more inpatient resources than children without ND. The prevalence of childhood disability is estimated to be 7.3% (CI 6.9–7.7%) [10]. In our sample hospital population, children with one or more ND accounted for 15.3% of the inpatient population, 17.7% of total hospital admission episodes and 27.8% of total inpatient days. The results in our study follows the pattern of studies in the US: children with ND accounted for 13.5% of hospitalisations (total number of hospital admissions), 25% of bed days and 29.0% of hospital charges, in children’s hospitals [2]. These figures are not dissimilar to our results. The results also fit with the idea that a small group within the neurological disabled population accounts for a large proportion of healthcare resources [3, 4]. This group was identified as children with more than one ND which accounted for 4.0% of the inpatient population, 5% of hospital admission episodes and 11.5% of inpatient days (Fig. 1). There was no statistically significant difference in hospital admissions between children with more than one ND and children with one ND. However, children with more than one ND accounted for statistically significantly more inpatient days than children with one ND, a pattern seen medicine, specialties and surgery (Fig. 2). This disparity in resource use was not as clear when analysing the different categories of ND separately. Our results show that inpatient resource use is not uniform across categories of ND. Post-hoc analysis showed that both children with more than one ND and children with genetic, chromosomal and syndromic disorders used more inpatient resources than children without ND in terms of hospital admissions and inpatient days. However, children with more than one ND did not use significantly more inpatient resources than children with one ND, as one might expect, when split into the different categories of ND. One reason for this may be large variance in data due to the small sample sizes of each category of ND. A future study, with a larger sample inpatient population could prospectively look at inpatient resource use by different neurological disability.

It is important to stress that not all inpatient resource use is negative. Hospital admissions allow the best care to be provided for patients that could not have been provided in the community. However, hospitalisations can be traumatising to both children and their families, especially in younger and more severely ill children [12]. The adverse effects of hospitalisations include psychological trauma, medical errors and nosocomial infections [13]. Given that children with ND have more frequent hospitalisations and tend to have longer inpatient stays, it could be argued that this hospital population group is at greater risk of these adverse effects. The need for proactive strategies to reduce avoidable hospitalisations is even more important for children with ND. One possible strategy may be improvements in community care. Frequent admissions to hospitals could indicate a lack of care continuity [5]. A community team would be ideally placed to help better parental education regarding medication to increase adherence, avoiding disease triggers, and liaise with outpatient follow-up teams.

To date, little research of this kind has been done in the UK. This data should assist management decision making. This and any further research that follows, should provide an evidenced-based platform for service planning. For example, with coding so closely linked to hospital funding, there is scope for an improved system. The ideal system would allocate more funding to children with more neurological disabilities for the same illness or procedure, due to their longer inpatient stays. Other effective methods for ensuring that the needs of the most complex patients are met may be a regional ND register, or an “electronic health passport” [5]. This is based on the Australian model where new cases of cerebral palsy are to the Victorian Cerebral Palsy Register as soon as a diagnosis is made [6]. Linkage to the state’s hospital admissions databased allowed admissions and length of stays to be tracked and better inform the healthcare providers.

### Limitations

Setting a relatively short time frame, we were able to generate a hospital population sample of over 900 inpatients. Despite only looking at three months of data, we were still able to find a statistically significant differences in hospital admissions and total inpatient days. These findings are unlikely to be a chanced localised effect and we hypothesise that these differences can also be found at a national level. Further investigation should attempt to replicate these analysis over a period of 12 months or more to negate any seasonal trends in disease presentation.

## Conclusion

The percentage of children identified with ND in our sample hospital population was over double the estimate of the disability prevalence in the UK. Children with ND had more frequent hospital admission episodes, and longer inpatient stays. We identified a smaller group within this population, with arguably more complex neurological disabilities, children with more than one ND. This study adds to the existing literature, showing how complexity of neurological disability can influence inpatient resource use in the UK. Whilst not all inpatient resource use is negative, this data can be helpful to involved in service planning to develop pro-active strategies that minimises any unnecessary hospital admission. More frequent hospital admissions and longer inpatient stays may place children with ND at greater risk of the adverse effects of hospitalisations. We recommend further investigations looking at each the effects of the different categories of ND on inpatient resource use, and repeat of this study at a national level and over a longer period of time.

## Abbreviation

ND: neurological disabilities
